# Toxic Diatom Aldehydes Affect Defence Gene Networks in Sea Urchins

**DOI:** 10.1371/journal.pone.0149734

**Published:** 2016-02-25

**Authors:** Stefano Varrella, Giovanna Romano, Susan Costantini, Nadia Ruocco, Adrianna Ianora, Matt G. Bentley, Maria Costantini

**Affiliations:** 1 Department of Biology and Evolution of Marine Organisms, Stazione Zoologica Anton Dohrn, Villa Comunale, Napoli, Italy; 2 Department of Integrative Marine Ecology, Stazione Zoologica Anton Dohrn, Villa Comunale, Napoli, Italy; 3 CROM, Istituto Nazionale Tumori “Fondazione G. Pascale,” IRCCS, Napoli, Italy; 4 Faculty of Science and Technology, Bournemouth University, Talbot Campus, Poole, United Kingdom; Laboratoire de Biologie du Développement de Villefranche-sur-Mer, FRANCE

## Abstract

Marine organisms possess a series of cellular strategies to counteract the negative effects of toxic compounds, including the massive reorganization of gene expression networks. Here we report the modulated dose-dependent response of activated genes by diatom polyunsaturated aldehydes (PUAs) in the sea urchin *Paracentrotus lividus*. PUAs are secondary metabolites deriving from the oxidation of fatty acids, inducing deleterious effects on the reproduction and development of planktonic and benthic organisms that feed on these unicellular algae and with anti-cancer activity. Our previous results showed that PUAs target several genes, implicated in different functional processes in this sea urchin. Using interactomic Ingenuity Pathway Analysis we now show that the genes targeted by PUAs are correlated with four HUB genes, *NF-κB*, *p53*, *δ-2-catenin* and *HIF1A*, which have not been previously reported for *P*. *lividus*. We propose a working model describing hypothetical pathways potentially involved in toxic aldehyde stress response in sea urchins. This represents the first report on gene networks affected by PUAs, opening new perspectives in understanding the cellular mechanisms underlying the response of benthic organisms to diatom exposure.

## Introduction

Marine organisms are constantly exposed to environmental stimuli and natural and/or dissolved anthropogenic compounds, including both physical (e.g. cold, heat and osmotic condition) and chemical (e.g. endocrine disruptor chemicals and hydrocarbons) stressors [1]. Organisms may react to these stressors by activating a series of cellular defence systems, by changing gene expression levels and altering interactions among genes [2]. Studying changes in expression levels is straightforward, but examining the extent to which cells rewire gene network connections is more difficult [3]. Knowledge of these gene interactions provides a more comprehensive view of cellular responses to stressors and is important for the development of interventions that improve responses to perturbations. Genes that are induced during stress protect cells not only through the production of important metabolic proteins (functional proteins) but also by regulating signal transduction genes in the stress response [4].

Natural toxins can also represent a major source of stress for marine organisms. Examples include algal neurotoxins that can cause mass mortalities in fish, sea birds and marine mammals. Although diatom-derived cytotoxic compounds are less toxic than neurotoxins, some induce negative effects on grazers, including reproductive failure, abortions and abnormal development. Diatoms are eukaryotic unicellular plants and are among the dominant photosynthetic organisms in the world’s oceans. They are considered essential in the transfer of energy through marine food chains including important fisheries. Several studies, however, have shown that some diatom species produce secondary metabolites, collectively termed oxylipins (e.g. polyunsaturated aldehydes or PUAs) and other products, deriving from the oxidation of fatty acids with negative effects on copepods [5,6], cladocerans [7], sea urchins [8], sea stars [9,10], polychaete worms and ascidians [11,12]. The first molecular studies on the effects of PUAs on the sea urchin, *Paracentrotus lividus*, were reported only very recently [13–15]. These studies showed that PUAs induced teratogenesis (i.e. developmental malformations) in a dose dependent manner. Moreover, the expression levels of thirty-one genes, having a key role in a broad range of functional responses, such as development, differentiation and detoxification processes, were followed by real-time qPCR to identify potential target genes [15]. Our findings revealed that the expression levels of a large number of genes were modulated by the PUAs decadienal, heptadienal and octadienal. These molecular results supported morphological findings that revealed that the majority of malformations affected the skeleton, the developmental plan and differentiation of sea urchin embryos [15]. In fact, several genes belonging to the skeletogenic, developmental and differentiation classes were affected by PUAs.

The aim of this work was to further explore the toxic effects of these PUAs on gene expression in the sea urchin *P*. *lividus*. Firstly, we treated embryos with increasing concentrations of the three PUAs in order to study a possible dose-dependent response of genes highlighted in Varrella et al. [15]. Then, we performed an interactomic analysis on modulated genes to understand if these were inter-correlated and if they were involved in specific gene networks.

## Materials and Methods

### Ethics statement

*Paracentrotus lividus* (Lamarck) sea urchins were collected from a location that is not privately-owned or protected in any way, according to Italian legislation of the Marina Mercantile (Decreto del Presidente della Repubblica DPR 1639/68, 09/19/1980 confirmed on 01/10/2000). The field studies did not involve endangered or protected species. All animal procedures were in compliance with the guidelines of the European Union (Directive 609/86).

### Gamete collection, exposure to aldehydes and embryo cultures for RNA extraction, cDNA synthesis

Adult *P*. *lividus* were collected during the breeding season by scuba-diving in the Gulf of Naples, transported in an insulated box to the laboratory within 1 h of collection and maintained in tanks with circulating sea water until required for experimentation. Sea urchins were injected with 2M KCl through the peri-buccal membrane to bring about the release of gametes. Following shedding, eggs were washed with filtered sea water (FSW) and kept in FSW until use. Concentrated (dry) sperm was collected, and kept undiluted at +4°C until use.

Before fertilization about 30,000 eggs in 200 mL of FSW were incubated at room temperature for 10 min in the presence of different concentrations of the three PUAs: 2*-trans*,4-*trans*-decadienal at 1.0, 1.3, 1.6, 2.0, 2.3 μM (similar to the concentrations tested in Varrella et al., 2014); 2-*trans*,4-*trans*-heptadienal at 2.0, 2.5, 3.0, 5.5, 6.0 μM; 2-*trans*,4-*trans*-octadienal (Sigma-Aldrich, Milan, Italy) at 2.5, 4.0, 4.5, 7.0, 8.0 μM; the controls were performed in FSW without PUAs. PUAs were diluted in methanol, considering a methanol to FSW ratio of 10 μL:1 mL, so as to avoid interference with embryo development. Controls were also performed in FSW and in FSW in the presence of methanol without PUAs. Eggs were then fertilized, utilising sperm-to-egg ratios of 100:1 for both controls and treated embryos. Fertilized eggs were kept at 20°C in a controlled temperature chamber on a 12 h:12 h light:dark cycle (fluorescent white lamps, light intensity 150 μmol photon m^-2^ sec^-1^) and collected at different developmental times. Samples were collected at 5, 9, 24 and 48 hours post fertilization (hpf) by centrifugation at 1800 relative centrifugal force for 10 min in a swing out rotor at 4°C. The pellet was washed with phosphate buffered saline and then frozen in liquid nitrogen and kept at −80°C. Experiments were conducted in triplicate using three egg groups collected from three different females. Embryos were fixed in formaldehyde (4% in FSW) and then observed under a light microscope (Zeiss Axiovert 135TV, Carl Zeiss, Jena, Germany).

Total RNA was extracted from each developmental stage using TRIzol (Invitrogen, Life Technologies, Carlsbad, CA, USA) according to the manufacturer’s instructions. Extraction with chloroform/isoamyl alcohol (24:1) was performed following RNA precipitation by addition of glycogen and isopropyl alcohol. Contaminating DNA was degraded by treating each sample with a DNase RNase-free kit (Roche, Milan, Italy) according to the manufacturer’s instructions. The amount of total RNA extracted was estimated by the absorbance at 260 nm and the purity by 260/280 and 260/230 nm ratios, by a NanoDrop spectrophotometer (ND-1000 UV-Vis Spectrophotometer; NanoDrop Technologies, Wilmington, DE, USA). The integrity of RNA was evaluated by agarose gel electrophoresis. Intact rRNA subunits (28S and 18S) were observed on the gel indicating minimal degradation of the RNA. For each sample, 600 ng of total RNA extracted was retrotranscribed with an iScript cDNA Synthesis kit (Bio-Rad, Milan, Italy), following the manufacturer’s instructions. Synthetized cDNA was used in real-time qPCR experiments without dilution.

To evaluate the efficiency of cDNA synthesis, a PCR was performed with primers of the reference gene, ubiquitin. The reaction was carried out on the C1000 Touch Thermal Cycler GeneAmp PCR System 9700 (Applied Biosystem, Monza, Italy) in a 30 μL final volume with 3 μL 10× PCR reaction buffer (Roche, Milan, Italy), 3 μL 10× 2 mM dNTP, 1 μL 5 U/μL Taq (Roche, Milan, Italy), 100 ng/μL of each oligo, template cDNA and nuclease free water to 30 μL. The PCR program consisted of a denaturation step at 95°C for 5 min, 35 cycles at 95°C for 45 s, 60°C for 1 min and 72°C for 30 s and a final extension step at 72°C for 10 min.

### Gene expression by Real-Time qPCR

The expression level of thirty-one genes was detected at five increasing concentrations of the three PUAs (see Results section). Moreover, in this work we also analyzed four new genes (S1 Table): nuclear factor kappa-light-chain-enhancer of activated B cells (*NF-κB*) [16], tumor protein p53 (*p53*), cadherin-associated protein (catenin) delta 2 (*Ctnnd2*), hypoxia inducible factor 1-alpha (*HIF1A*). *NF-κB* was studied by using the primers suggested in Russo et al. [16]. Since *p53*, *Ctnnd2* and *HIF1A* have never been studied in *P*. *lividus* and the gene sequences were not available, specific primers were designed on the basis of nucleotide sequences of these genes from *Strongylocentrotus purpuratus*, retrieved from SpBase (http://www.spbase.org/SpBase/; see Table 1).

**Table 1:** Accession numbers, primer sequences and lengths of PCR amplified fragments are reported for the four new genes analyzed.

Gene	Acronym	Acc. Number	Primer	Sequence (5' = >3')	PCR fragment (bp)
***Nuclear factor kappa-light-***	*NF-κB*		Pl-NF-kB_F	TCCCATGGAGGACTGCCGTGTCA	**116**
***chain-enhancer of activated B cells***			Pl-NF-kB_R	TCGTTGGTTACCAAGGAGACCACA	
***(Russo et al*., *2013)***					
***Tumor protein p53***	*p53*	SPU_023158.1	Sp_p53_F1	GCGTTGGTGGATCATACTGG	163
			Sp_p53_R1	GATCTTGGTCTGAGCGTAGTG	
***Cadherin-associated protein***	*Ctnnd2*	SPU_001161.1	Sp_Catenin_F1	GGATACTCAATCAAGATCACAAC	229
***(catenin) delta 2***			Sp_Catenin_R1	CTCTGACAGTACAATGAGATATGG	
***Hypoxia inducible factor***	*HIF1A*	SPU_030140.1	Sp_HIF1A_F1	CGATAGAAGAGATCATCGACTC	158
***1-alpha***			Sp_HIF1A_R1	GTAGTCGTAGATGCTCTGGC	

The amplified fragments using a Taq High Fidelity PCR System (Roche, Milan, Italy) were purified from agarose gel using the QIAquick Gel Extraction kit (Qiagen, Milan, Italy), and the specificity of the PCR products for *p53*, *Ctnnd2* and *HIF1A* genes were checked by DNA sequencing.

PCR amplifications were performed in a ViiATM7 Real Time PCR System (Applied Biosystems, Monza, Italy) thermal cycler using the following thermal profile: 95°C for 10 min, one cycle for cDNA denaturation; 95°C for 15 s and 60°C for 1 min, 40 cycles for amplification; 72°C for 5 min, one cycle for final elongation; one cycle for melting curve analysis (from 60°C to 95°C) to verify the presence of a single product. Each assay included a no-template control for each primer pair. To capture intra-assay variability, all real-time qPCR reactions were carried out in triplicate. Fluorescence was measured using ViiATM7 software (Applied Biosystems, Monza, Italy). The expression of each gene was analysed and internally normalized against ubiquitin as a reference gene, using REST software (Relative Expression Software Tool, Weihenstephan, Germany) based on the Pfaffl method [17,18]. The two-fold expression level was therefore chosen as the threshold for significance of target genes. However, to validate our results, a statistical analysis was also performed using GraphPad Prism version 4.00 for Windows (GraphPad Software, San Diego California USA).

### Interactomic analysis

The network analysis was performed by Ingenuity Pathway Analysis Version 7.1 (IPA, Ingenuity Systems, Inc., Redwood City, CA, United States) to identify relationships between relevant *P*. *lividus* genes analyzed in this work, on the basis of associated functions and data mining from experimental studies reported in the literature.

Ingenuity Pathway Analysis (IPA) is a system that transforms a list of genes (with or without accompanying expression information) into a set of relevant networks based on extensive records maintained in the Ingenuity Pathways Knowledge Base (IPKB). This knowledge base has been abstracted into a large network, called the Global Molecular Network, composed of thousands of genes and gene products that interact with each other. The network is displayed graphically as nodes (genes) and edges (the biological relationships between nodes). Two genes are connected if there is a path in the network between them, i.e., a series of genes and edges that connect one gene to another. HUB nodes are viewed as important nodes in a network: they are nodes with the largest degrees, i.e., nodes that share the largest number of connections with the other nodes.

Because sea urchin genes are not annotated in the IPA database, we used the name of the human orthologous genes to search the *P*. *lividus* genes. In fact, species supported with full content in IPA are human, mouse and rat.

## Results

### Dose-dependence effects of PUAs on gene expression

Developing embryos of *P*. *lividus* were incubated in the presence of five increasing PUAs concentrations (decadienal at 1.0, 1.3, 1.6, 2.0, 2.3 μM; heptadienal at 2.0, 2.5, 3.0, 5.5, 6.0 μM; octadienal at 2.5, 4.0, 4.5, 5.0, 7.0, 8.0 μM). The concentrations for decadienal were the same as those used in Marrone et al. [14]; since the effects induced by heptadienal and octadienal were not strong as with decadienal, we tested concentrations of heptadienal and octadienal that produced the same percentage of abnormal plutei as in the case of decadienal from Marrone et al. ([14]; 5%, 10%, 35%, 50% and 70%; see also Fig 2 in ref. [15]), so as to have comparable results with the three PUAs. Samples were collected at 5, 9, 24 and 48 hpf, corresponding to the stages of early blastula, swimming blastula, prism and pluteus.

**Fig 1:**
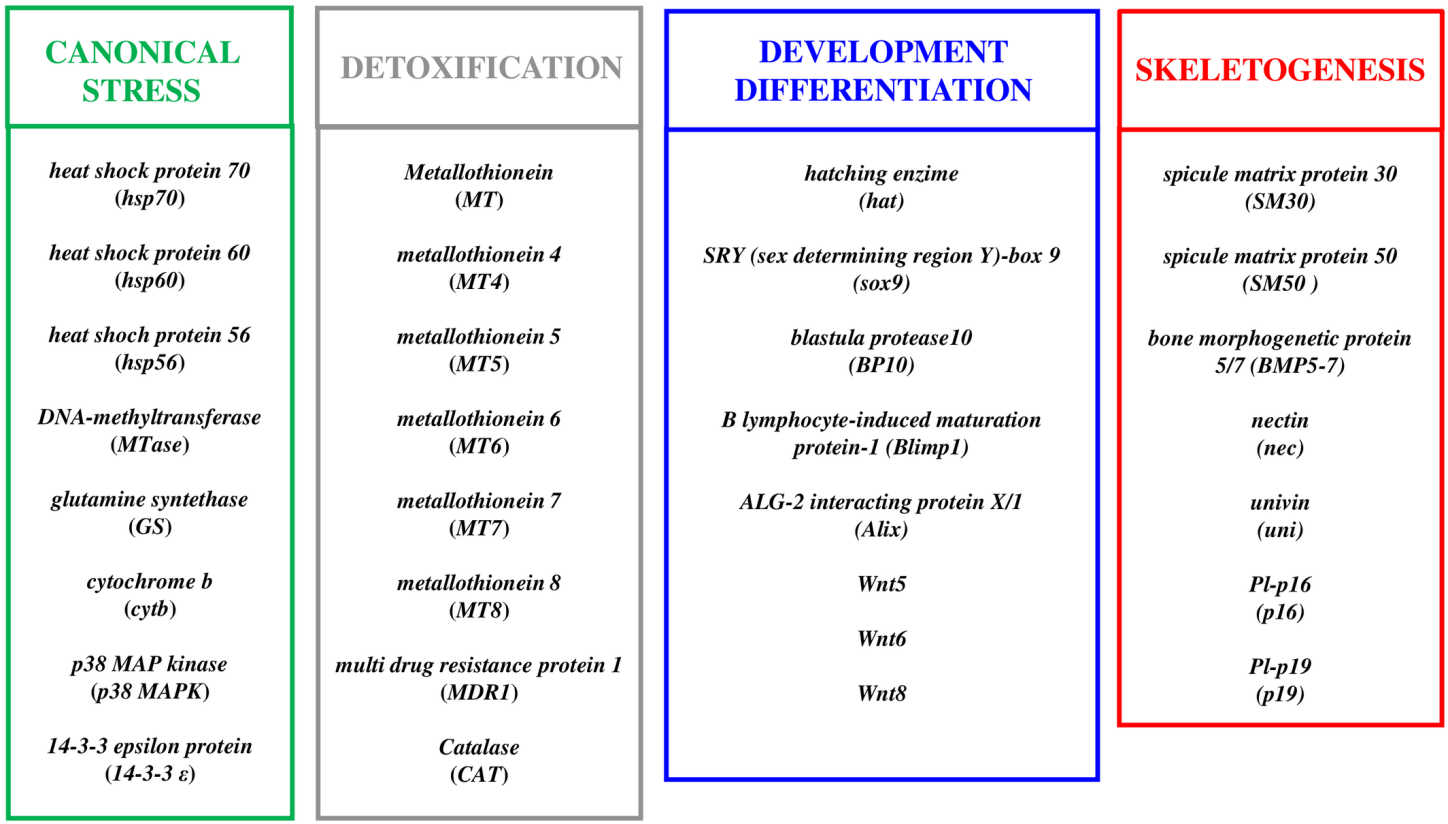
Gene functional classes. The scheme indicates the four functional classes of genes used to which the thirty one genes analyzed in the present study belong: canonical stress genes, genes involved in detoxification processes, genes involved in developmental and differentiation processes and skeletogenic genes.

We studied the possible dose-dependent effects on the thirty one genes analyzed in our previous work only at the teratogenic concentrations of the three PUAs (decadienal 1.6 μM, heptadienal 3.0 μM, octadienal 4.5 μM), producing about 35% of abnormal plutei [13–15]. These genes belong to four different classes: eight canonical stress genes, eight genes involved in detoxification processes, eight genes involved in developmental and differentiation processes, and seven skeletogenic genes, as reported in Fig 1.

**Fig 2:**
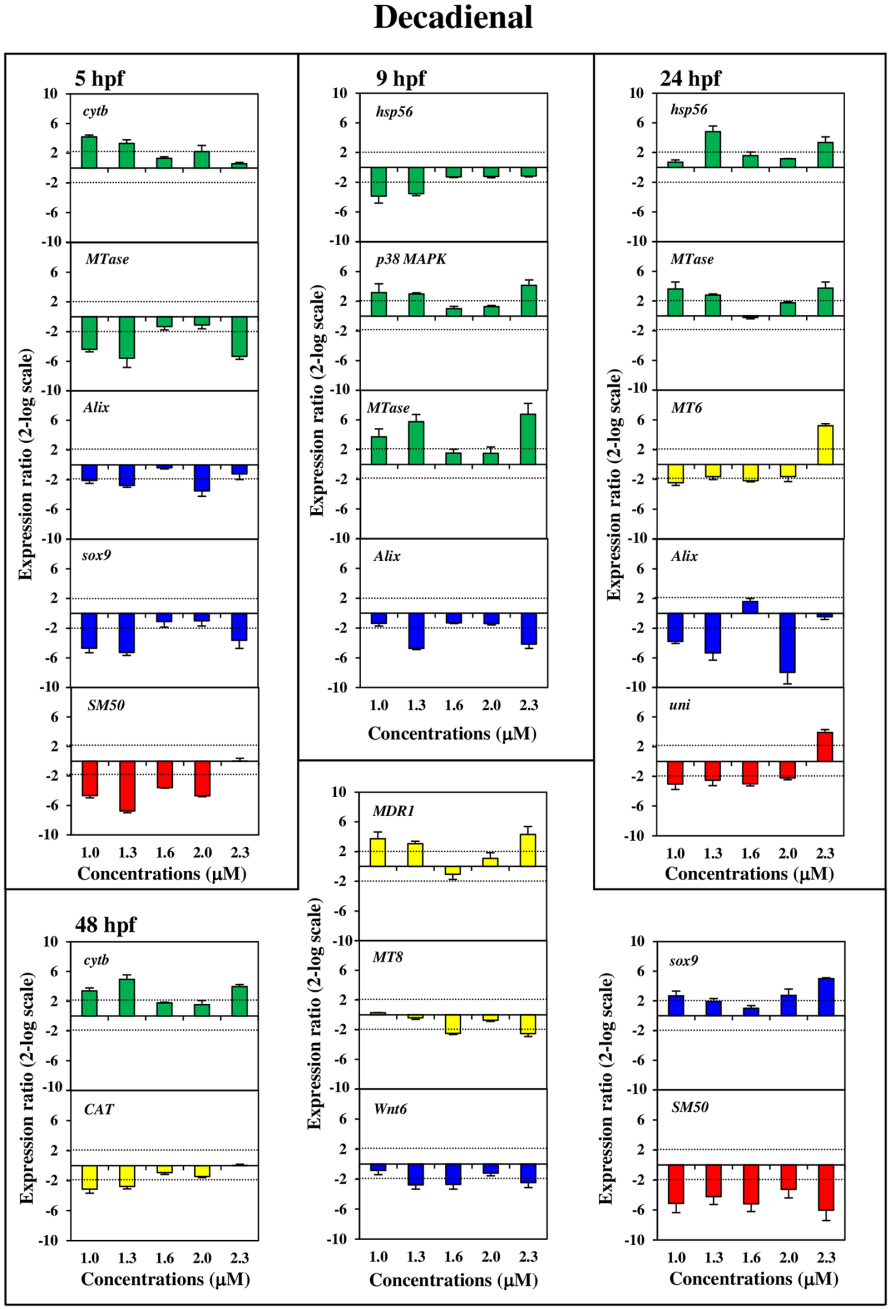
Dose-dependent variation of gene expression levels induced by decadienal for the genes analyzed. Histograms show dose-dependent variations in expression levels of decadienal modulated genes. Samples incubated with increasing decadienal concentrations (1.0, 1.3, 1.6, 2.0, 2.3 μM) were collected at different stages of development: early blastula (5hp), swimming blastula (9hpf), prism (24hpf) and pluteus (48 hpf) Data are reported as a fold difference (mean ± SD), compared to the control embryos in sea water without decadienal. Fold differences greater than ±2 (see the dotted horizontal guide lines at the values of +2 and −2) were considered significant. A colour code has been used in the histograms to distinguish the four functional classes of genes: green for stress genes, grey for genes involved in detoxification processes, blue for genes involved in developmental and differentiation processes, and red for skeletogenic genes.

**Fig 3:**
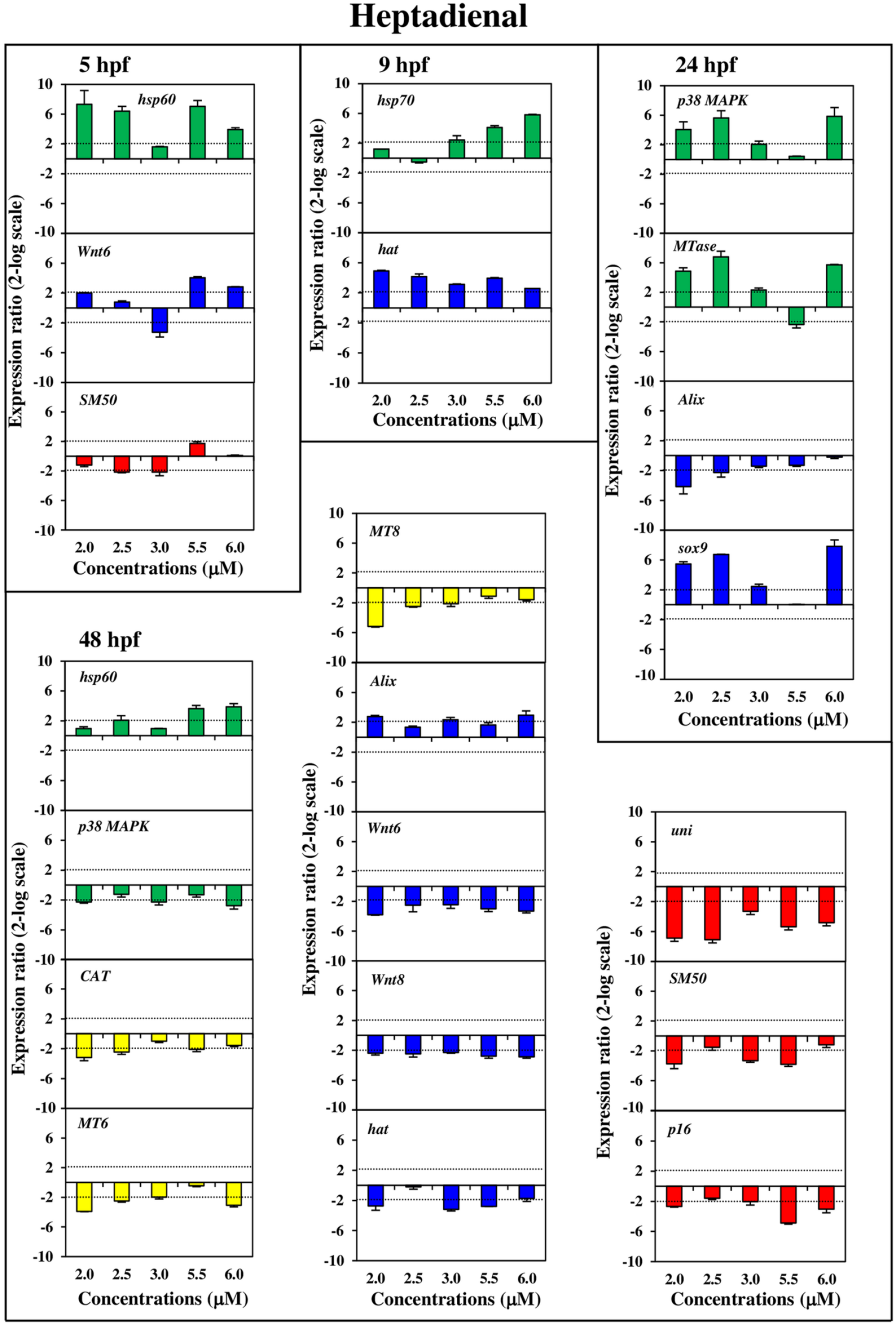
Dose-dependent variation of gene expression levels induced by heptadienal for the genes analyzed. Histograms show dose-dependent variations in expression levels of heptadienal modulated genes. Samples incubated with increasing heptadienal concentrations (2.0, 2.5, 3.0, 5.5, 6.0 μM) were collected at different stages of development: early blastula (5hp), swimming blastula (9hpf), prism (24hpf) and pluteus (48 hpf). For further details see also legend to Fig 2.

**Fig 4:**
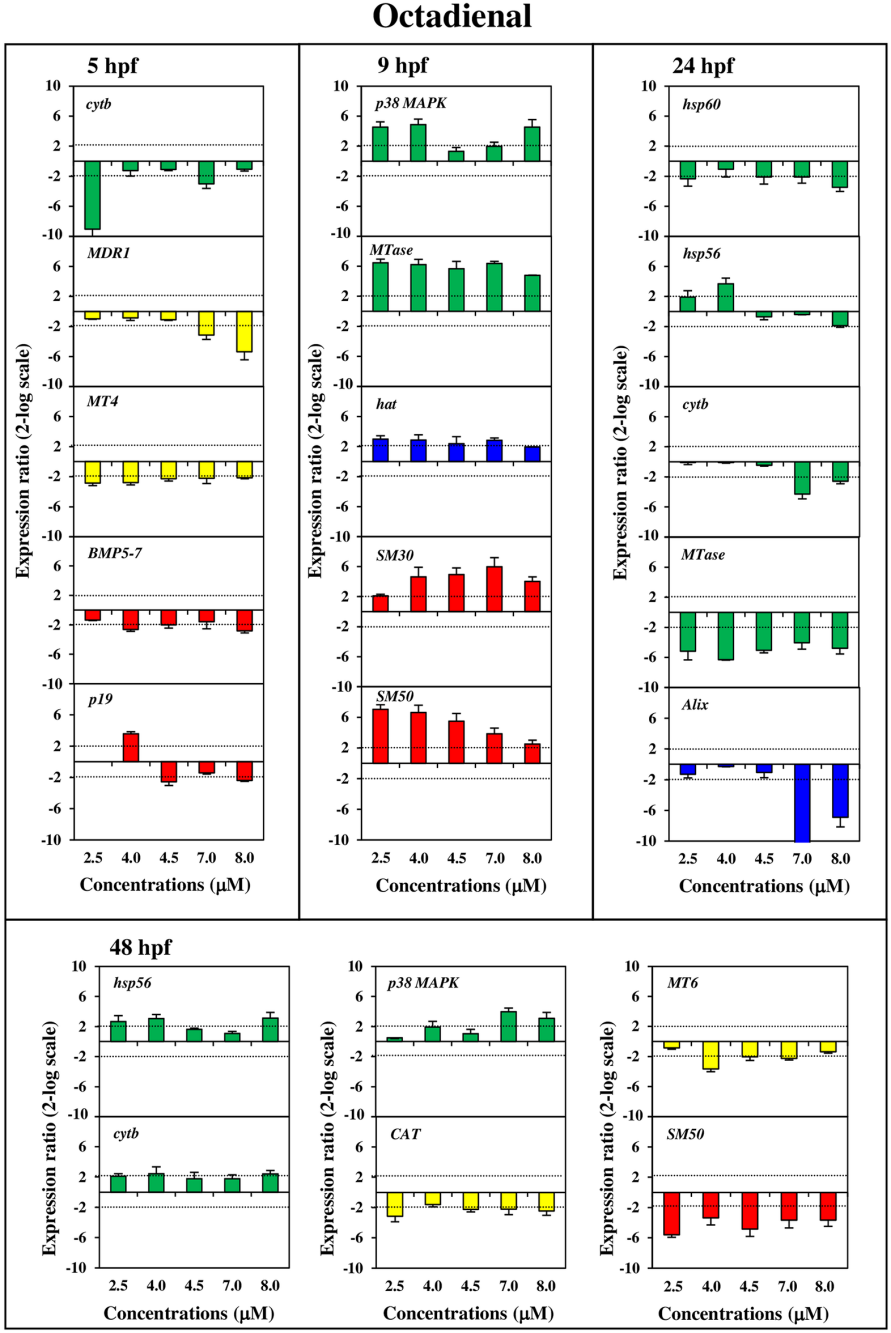
Dose-dependent variation of gene expression levels induced by octadienal for the genes analyzed. Histograms show dose-dependent variations in expression levels of octadienal modulated genes. Samples incubated with increasing octadienal concentrations (2.5, 4.0, 4.5, 5.0, 7.0, 8.0 μM) were collected at different stages of development: early blastula (5hp), swimming blastula (9hpf), prism (24hpf) and pluteus (48 hpf). For further details see also legend to Fig 2.

Our results showed a PUAs dose- and stage-dependent effect at the gene level for most of the analysed genes of all four functional classes. The histograms with the relative expression ratios of the analyzed genes detected by Real Time qPCR (with respect to the control without PUAs) are reported in Figs 2, 3 and 4. The exact values of x-fold changes for all the genes are reported in S2 Table.

1. Stress genes
  All stress genes (see green histograms in Figs 2, 3 and 4) were targeted at the different PUAs concentrations and also at the different developmental stages, except for the *14-3-3 ε* gene. More specifically:
  - *hsp70* was up-regulated only after heptadienal treatment at concentrations of 3.0, 5.5 and 6.0 μM at 9 hpf;
  - *hsp60* was up-regulated with heptadienal at 5 hpf (at 2.0, 2.5, 5.5 and 6.0 μM) and at 48 hpf (at 2.5, 5.5 and 6.0 μM) and down-regulated with octadienal at 24 hpf (at 2.5, 4.5, 7.0 and 8.0 μM);
  - *hsp56* was down-regulated at 9 hpf (at 1.0 and 1.3 μM) and up-regulated at 24 hpf (at 1.3 and 2.3 μM) after decadienal treatment; octadienal induced an increase in expression levels at 24 hpf (at 2.5 and 4.0 μM) and at 48 hpf (at 2.5, 4.0 and 8.0 μM);
  - *MTase* was targeted by the three PUAs: decadienal induced a down-regulation at 5 hpf and up-regulation at 9 and 24 hpf (at 1.0, 1.3 and 2.3 μM); heptadienal upregulated only at 24 hpf (at 2.0, 2.5 and 6.0 μM); octadienal induced an up-regulated at 9 hpf and down-regulation at 24 hpf at all concentrations tested;
  - *cytb* was upregulated by decadienal at 5 hpf (at 1.0, 1.3 and 2.3 μM) and at 48 hpf (at 1.0, 1.3 and 2.3 μM) but remained at the basal level with heptadienal. This gene was down-regulated by octadienal at 5 hpf (at 2.5 μM and especially at 7.0 μM), at 24 hpf (at 7.0 and 8.0 μM), and at 48 hpf at all the concentrations tested;
  - *p38 MAPK* increased in expression levels at 9 hpf with decadienal (1.0, 1.3 and 2.3 μM); heptadienal induced increase of expression levels at 24 hpf with (at 2.0, 2.5 and 6.0 μM) but decreased at 48 hpf (at 2.0, 3.0 and 6.0 μM); increase of expression levels was also observed with octadienal at 9 hpf (at 2.5, 4.0 and 8.0 μM) and at 48 hpf (at 7.0 and 8.0 μM).
2. Detoxification genes
  For these genes the dose-dependent effects was closely related to the developmental stages (see yellow histograms in Figs 2, 3 and 4):
  - *MT4* was down-regulated by octadienal at 5 hpf, with a decrease in expression levels at all concentrations tested;
  - *MT6* was down-regulated by decadienal at 24 hpf (at 1.0, 1.6) whereas with heptadienal and octadienal this gene was up-regulated at 48 hpf (heptadienal 2.0, 2.5, 3.0 and 6.0 μM; octadienal 4.0, 4.5 and 7.0 μM);
  - *MT8* was targeted at 48 hpf only after treatment with decadienal (at 1.6 and 2.3 μM) and with heptadienal (at all the concentrations tested, except for 5.5 μM);
  - *MDR1*showed dose-dependent effects with decadienal at 48 hpf (at 1.0, 1.3 and 2.3 μM) and octadienal at 5 hpf (at 7.0 and 8.0 μM);
  - *CAT* was down-regulated at 48 hpf by decadienal (at 1.0 and 1.3 μM) and octadienal (at 2.5, 4.5, 7.0 and 8.0 μM).
3. Developmental and differentiation genes
  Developmental and differentiation genes were all affected by the three PUAs (see blue histograms in Figs 2, 3 and 4):
  - *hat* showed a very strong dose-dependent effect with a significant increase in gene expression after treatment with heptadienal at all concentrations tested; the effect was less evident with octadienal at 9 hpf whereas a decrease was observed with heptadienal at 48 hpf (at 2.0, 3.0, 5.5 μM);
  - *sox9* showed a strong dose-dependent effect with decadienal, decreasing at 5 hpf (at 1.0, 1.3 and 2.3 μM) and increasing at 48 hpf (at 1.0, 1.3, 2.0 and 2.3 μM) compared to the controls; heptadienal induced a very significant up-regulation at 24 hpf (at 2.0, 2.5 and 6.0 μM);
  - *Alix*, showed a decrease in expression levels with the three PUAs (decadienal at 5 hfp at 1.0, 1.3 and 2.3 μM, and 9 hpf at 1.3 and 2.3 μM; heptadienal at 24 and 48 hpf; octadienal at 24 hpf);
  - *Wnt6* was switched on by decadienal at 48 hpf (down-regulated at 1.3, 1.6 and 2.3 μM) whereas was activated by heptadienal at 5 hpf (down-regulated at 3.0 μM and up-regulated at 2.0, 5.5 and 6.0 μM) and 48 hpf (down-regulated at all concentrations tested);
  - *Wnt8* showed a dose-dependent effect only with heptadienal at all concentrations tested.
4. Skeletogenic genes
  The gene expression of these genes resulted in PUA-specific effects (see red histograms in Figs 2, 3 and 4):
  - *SM30* showed a strong dose-dependent effect only with octadienal, with expression levels increasing at 9 hpf at all concentrations tested;
  - *SM50* showed strong dose-dependent effect with decadienal with a down-regulation in expression levels at 5 and 48 hpf at all concentrations tested; with heptadienal the effects were somewhat less evident; octadienal induced an up-regulation at 9 hpf and a down-regulation at 48 hpf at all concentrations tested;
  - *BMP5/7* was switched on specifically by octadienal at 5 hpf (from 4.0 to 8.0 μM);
  - *uni* was down-regulated by decadienal at 24 hpf (from 1.0 to 2.0 μM) and up-regulated at 2.3 μM; heptadienal induced a strong dose-dependent effect at 48 hpf (at all concentrations tested)
  - *p16* was down-regulated only by heptadienal at 48 hpf (at 2.0, 3.0, 5.5 and 6.0 μM);
  - *p19* expression levels increased (at 4.0 μM) and decreased (at 4.5 and 8.0 μM) expression level with octadienal at 5 hpf.

**Fig 5:**
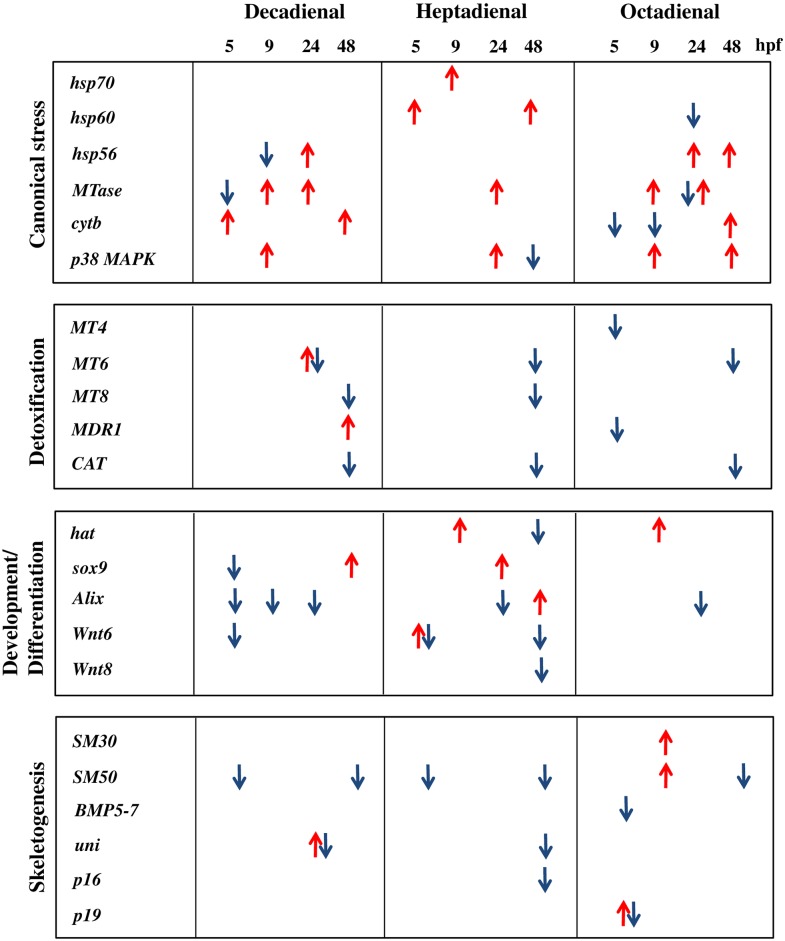
Synopsis of dose-dependent gene expression of genes analyzed. Patterns of dose-dependent up- (red arrows) and down-regulation (blue arrows) of the four classes of genes in the sea urchin, *P. lividus*, in the presence of the PUAs decadienal, heptadienal and octadienal. Genes with two arrows are up- and down-regulated at different concentrations. The arrows correspond to fold differences greater than ±2, considered significant levels of down- or up-regulation.

A synopsis showing the patterns of dose-dependent up- and down-regulation of different classes of genes is shown in Fig 5.

### Network analysis and RT qPCR of HUB genes

**Fig 6:**
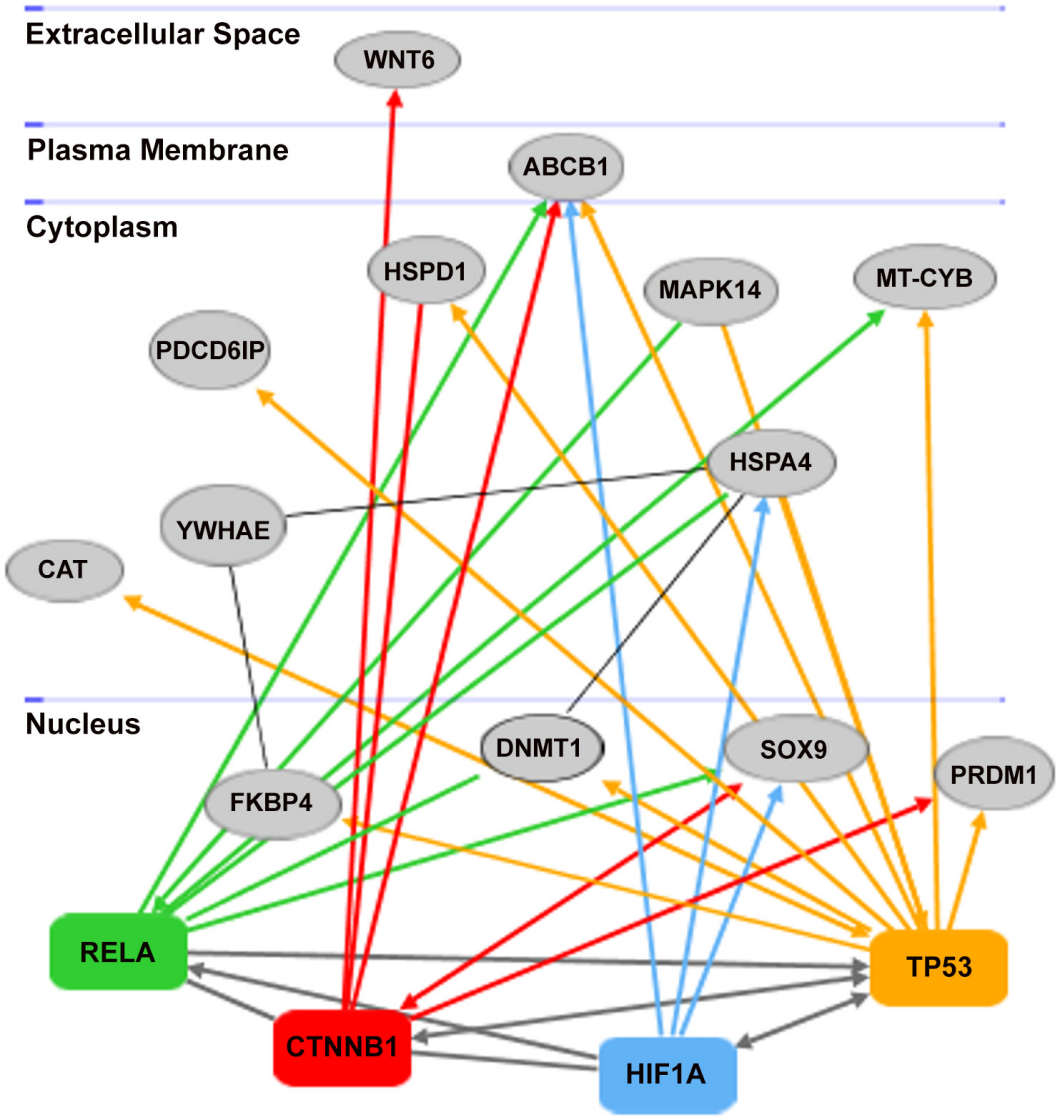
Interactomic analysis by Ingenuity Pathway Analysis (IPA) software. The network is displayed graphically as nodes (genes) and edges (the biological relationships between nodes). HUB nodes, genes that share the largest numbers of connections with other genes, are indicated by symbols of different colors: *RELA* in green; *CTNNB1* in red, *HIF1A* in light blue and *TP53* in yellow. The biological relationships between HUB nodes and the other significant genes are indicated by coloured arrows (indicating that a molecule modulates the expression of another), according to the colours of the HUB to which they are connected. The connections between CTNNB1-HIF1A, CTNNB1-RELA are indicated by edges and not by arrowheads because the solid edges indicate direct relationships between molecules due to real chemical modifications and, hence, to formation of direct physical contacts. Interaction between HUB nodes are indicated with grey arrows. Genes associated with HUB genes are reported with grey symbols. For further details on IPA analysis see also Materials and Methods section.

Interactomic analysis indicated that there were four HUB genes (Fig 6): *RELA* (nuclear factor NF-kappa-B p65 subunit), *CTNNB1* (Catenin, Cadherin-Associated Protein, Beta 1), *HIF1A* (hypoxia inducible factor 1-alpha) and *TP53* (tumor protein p53). HUB nodes are viewed as important nodes in a network: they are nodes with the largest degrees, i.e., nodes that share the largest number of connections with the other nodes.

These four genes represent nodes having a large degree of interaction, indicating that they have connections with many other genes. None of these genes has been studied before in *P*. *lividus*, with the exception of *NF-κB* (of which *RELA* represents a subunit) [16,19]. Therefore, we used annotated genes from *S*. *purpuratus* (for more details see Methods section) and we analyzed tumor protein p53 (*p53*; corresponding to *TP53* in humans), cadherin-associated protein (catenin) delta 2 (*Ctnnd2* or *δ-2-catenin*, for human *CTNNB1*), and hypoxia inducible factor 1-alpha (*HIF1A*). Fig 3 shows the close functional association between HUB nodes and significant genes (see also Table 2): i) *RELA* gene interacts with *ABCB1*, *MAPK14*, *MT-CYB* and *HSPA4*; ii) *CTNNB1* interacts with *WNT6*, *HSPD1*, *ABCB1*, *SOX9* and *PRDM1*; iii) *HIF1A* interacts with *ABCB1*, *HSPA4* and *SOX9*; iv) *TP53* interacts with *FKBP4*, *CAT*, *PDCD6IP*, *HSPD1*, *ABCB1*, *MAPK14*, *MT-CYB* and *PRDM1*. HUB nodes also interacted between themselves. *YWHAE* gene is the only not directly connected with HUB genes, but it interacts with *HSPA4* (connected with *HIF1A*) and *FKBP4* (connected with *TP53*). All the genes associated with HUB nodes were targeted by the three PUAs, with the exception of *YWHAE* and *PRDM1*.

**Table 2:** The corresponding names of *P*. *lividus* and human genes are reported.

Gene name	*P. lividus*	Human
***Heat shock protein 70***	*hsp70*	*HSPA4*
***Heat shock protein 60***	*hsp60*	*HSPD1*
***heat shock protein 56***	*hsp56*	*FBKP4*
***cytochrome b***	*cytb*	*MT-CYB*
***14-3-3 epsilon protein***	*14-3-3 ε*	*YWHAE*
***p37 mitogen-activated protein kinase***	*p38 MAPK*	*MAPK14*
***DNA-methyltransferase 1***	*Mtase*	*DNMT1*
***SRY (sex determining region Y)-box 9***	*sox9*	*SOX9*
***ALG-2 interacting protein X/1***	*Alix*	*PDCD6IP*
***Blimp***	*Blimp*	*PRDM1*
***Wnt6***	*Wnt6*	*WNT6*
***Multi drug resistance protein 1***	*MDR1*	*ABCB1*
***Catalase***	*CAT*	*CAT*

**Fig 7:**
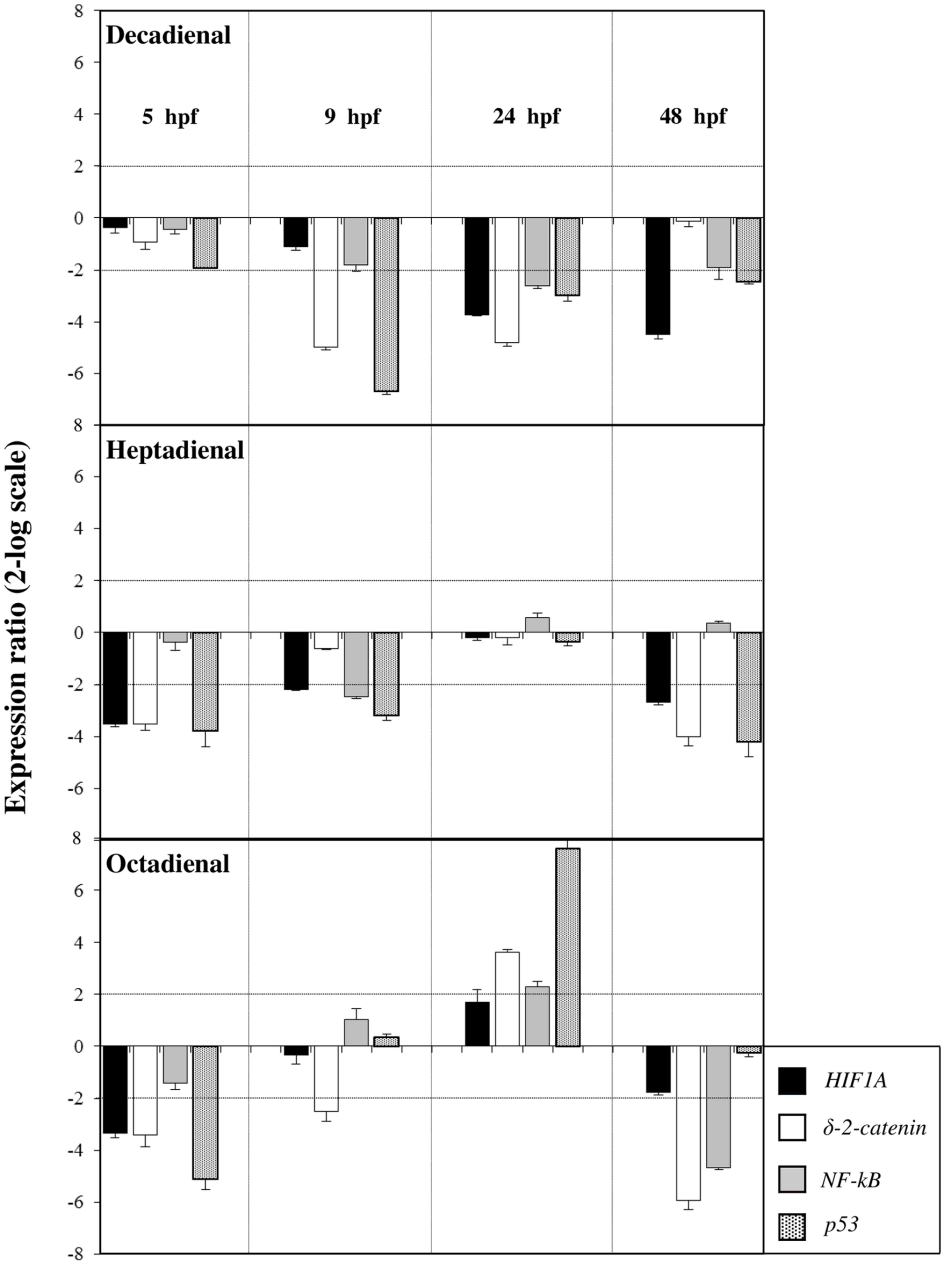
Gene expression level of HUB genes. The histograms show the differences in the expression levels of the four HUB genes followed by real-time qPCR, identified in *P*. *lividus*: *NF-κB*, *δ-2-catenin*, *HIF1A* and *p53*. Embryos were grown in the presence of decadienal, heptadienal and octadienal at teratogenic concentrations (1.6, 3.0 and 4.5 μM, respectively) and collected at different times of development (5, 9, 24 and 48hpf). Data are reported as a fold difference (mean ± SD), compared to the control, embryos in sea water without aldehydes. Fold differences greater than ±2 (see the dotted horizontal guide lines at the values of +2 and −2) were considered significant.

The expression levels of these four genes were followed by Real Time qPCR, to detect if they were targets of PUAs at teratogenic concentrations (decadienal 1.6 μM, heptadienal 3.0 μM and octadienal 4.5 μM). The histograms reported in Fig 7 show the relative expression ratios of these genes with respect to control embryos in FSW without PUAs. Only expression values greater that a two-fold difference with respect to the control were considered as significant.

At the early blastula stage (5 hpf), the expression levels of all four genes remained at basal levels and were comparable to the control after decadienal treatment. Interestingly, heptadienal and octadienal affected the expression levels of *HIF1A*, *δ-2-catenin* and *p53*. At the swimming blastula stage (9 hpf) decadienal and heptadienal down-regulated the gene *p53* (6.7- and 3.8 fold, respectively). The expression level of *δ-2-catenin* was significantly down-regulated by decadienal and octadienal (5.0- and 3.4-fold change, respectively). A down-regulation of *NF-κB* was recorded after treatment with heptadienal. At the prism stage (24 hpf) decadienal and octadienal differentially affected the expression levels of three genes: whereas decadienal down-regulated the expression levels of *δ-2-catenin*, *NF-κB* and *p53*. Octadienal up-regulated all these genes. At this developmental stage *HIF1A* showed a 3.7-fold decrease in expression level only with decadienal. At the pluteus stage (48 hpf) *HIF1A* was targeted by decadienal and heptadienal (4.5- and 2.7-fold decrease, respectively). Heptadienal and octadienal affected the expression levels of *δ-2-catenin* gene (4.0- and 5.9-fold decrease, respectively). *NF-κB* was targeted only after octadienal treatment (4.7-fold decrease). Finally, a down-regulation of the expression levels of *p53* was recorded for decadienal (2.4-fold decrease) and heptadienal (4.2-fold decrease).

A PUAs dose- and stage-dependent effect was also detected by Real Time qPCR at the gene level for these HUB genes (S2 Table for the corresponding values). Decadienal induced a significant dose-dependent effect specifically on *δ-2-catenin* and *p53* at 9 hpf, on *HIF1A*, *δ-2-catenin* and *p53* at 24 hpf and on *HIF1A* at 48 hpf (S1A Fig). In the case of heptadienal, the dose-dependent effect was detectable at 5 and 48 hpf for *HIF1A*, *δ-2-catenin* and *p53* (S1B Fig). Octadienal induced similar results to heptadienal with a dose-dependent effect at 5 hpf for *HIF1A*, *δ-2-catenin* (as well as at 48 hpf) and *p53* (S1C Fig). *NF-κB* was rarely activated at the five different concentrations tested (see as an example decadienal and heptadienal at 9 hpf, decadienal at 24 hpf).

## Discussion

Our previous results (reported in ref. [15]) showed that treatment with the three aldehydes induce similar malformations at the morphological level in sea urchin embryos. Interestingly, at the molecular level the three PUAs have very few common targets and specifically affect different classes of genes and at different developmental stages. These results suggest that PUAs may affect different physiological processes, considering that genes targeted by PUAs have a key role in a broad range of functional responses, such as stress, development, differentiation, skeletogenesis and detoxification processes (Fig 1). These findings are in according with the data reported by Sansone et al. (2014) [20] who also found that the same three PUAs triggered different cell signalling death pathways in the lung adenocarcinoma cell lines A549. Further investigations are needed to clarify why the three PUAs elicit differing changes in gene expression, considering that the fundamental reactive elements of the aldehyde molecules are the same (Michaels acceptor).

In our study we demonstrated a PUA dose-dependent effects on the expression of most genes, already switched on at low concentrations (decadienal 1.0 and 1.3 μM; heptadienal 2.0 and 2.5 μM; octadienal 2.5 and 4.0 μM), while the percentage of abnormal plutei is still low (as showed in the Figure 2 in ref. 15). More specifically, our study shows that some target genes respond differently to PUAs than others (see Figs 2, 3, 4 and 5 and S2 Table). This is the case for example for *SM50* after treatment with decadienal at 48 hpf (S1A Fig) and octadienal at 9 hpf (S1C Fig); *hsp60* at 5 hpf, *MTase* and *sox9* at 24 hpf and *uni* at 48 hpf after treatment with heptadienal (S1B Fig); *MTase* with octadienal at 9 and 24 hpf (S1C Fig); *δ-2-catenin* and *p53* at 9 hpf with decadienal (S1A Fig), *δ-2-catenin* and *HIF1A* with decadienal at 24 hpf (S1A Fig); *p53*, *δ-2-catenin* and *HIF1A* with heptadienal at 48 hpf (S1B Fig).

Some genes showed a dose-dependent variation in their expression levels at all concentrations tested, with the only exception of the highest concentrations (decadienal 2.3 μM; heptadienal 6.0 μM; octadienal 8.0 μM): for example the gene *SM50* at 5 hpf (see Fig 1) and *HIF1A* a 48 hpf (see S1A Fig) with decadienal (from 1.0 to 2.0 μM), the gene *δ-2-catenin* at 5 hpf with heptadienal (from 2.0 to 5.5 μM; see S1B Fig). Moreover, aldehyde treatments at teratogenic concentrations did not affect the expression level of some genes, the activation of which by various stress conditions is well-documented. This is the case of *p38 MAPK* that was a target gene only for heptadienal at 24 and 48 hpf; *Alix* at 48 hpf was targeted only by heptadienal; *MDR1* and *cytb* were not target genes after treatment with the three aldehydes. Different extracellular stimuli (including UVB irradiation, heat shock, high osmotic stress, pro-inflammatory cytokines and certain mitogens) trigger a stress-regulated protein kinase cascade culminating in activation of p38 MAPK through phosphorylation [21–25]. *MDR1* gene products are thought to play a role in the protection of organisms against toxic xenobiotics [26]; response to environmental stress was also reported for *cytb* [27]. No reports on the stress response of *Alix* gene have been reported, with the exception of Varrella et al. [15], who showed that this gene is modulated by heptadienal at 48 hpf. In the present study we recorded that the expression levels of these four genes were affected by the three PUAs treatments, confirming that these were markers of stress conditions but their induction required certain PUAs concentrations. An example of this is represented by *p38 MAPK* that was already activated at the lowest concentrations of heptadienal (2.0 and 2.5 μM; Fig 1) than the teratogenic concentration (4.5 μM) at 24 hpf and octadienal (2.5 and 4.0 μM; Fig 3) at 9 hpf.

In this study we show for the first time that the *Alix* gene responds to stress because its expression levels are targeted after decadienal treatment at 9 and 24 hpf at the lowest concentrations (1.3 and 1.0 μM). The same findings were also recorded with octadienal at the higher concentrations with high levels of variation of expression levels at the prism stage (10.8- and 6.9-fold at 7.0 and 8.0 μM, respectively). Decadienal and octadienal switched on the *MDR1* gene at the pluteus and blastula stages, respectively; decadienal switched on *cytb* at the highest concentrations at 5, 24 and 48 hpf; the same was also true for octadienal. These data suggest a very subtle adjustment to diatom-derived PUAs effects in developmental processes of sea urchin embryos. Moreover, our study provides the first evidence on the strong effects of heptadienal compared to other PUAs on sea urchins, supporting the data reported by Sansone et al. [20] on a human lung cancer cell line.

All *P*. *lividus* genes were analyzed by Ingenuity Pathway Analysis, which created a network on the basis of associated functions and data mining from experimental studies reported in the literature (Fig 6). It is important to consider that IPA software only supported vertebrate models with full content (for further details see also Materials and Methods Section), so gene interactions likely hold true in sea urchins as well as could be some differences. Our data indicate four HUB genes, including *NF-κB* (*RELA* represents a subunit of *NF-κB*), *p53* (corresponding to *TP53* in humans), *δ-2-catenin* (corresponding to human *CTNNB1*) and *HIF1A* in *P*. *lividus*. NF-kB is a protein complex that controls transcription of DNA and regulates the activities of many signalling pathways within the intracellular network, playing a role in immune response, inflammation, infection, oncogenesis and apoptosis, and in determining cellular responses to extracellular stimuli [28]. NF-κB is found in almost all animal cell types and is involved in cellular responses to stimuli such as stress, cytokines, free radicals, ultraviolet irradiation [16], oxidized LDL, and bacterial or viral antigens [29–32]. Moreover, this gene is a homo- or heterodimeric complex formed by the Rel-like domain-containing proteins RELA/p65, RELB, NFKB1/p105, NFKB1/p50, REL and NFKB2/p52 and the heterodimeric p65-p50 complex that appears to be the most abundant. The p53 protein is crucial in multicellular organisms, where it regulates the cell cycle and, thus, functions as a tumor suppressor, preventing cancer. The cell cycle checkpoint gene *p*53 allows a multicellular organism to repair or delete cells exposed to agents that cause DNA damage, like hypoxia, UVR, ROS or mutagens [33–36]. Up-regulation and expression of *p*53 allows DNA editing and repair to occur followed either by normal cell division [37] or apoptosis [38]. Moreover, p53 promotes proteasomal degradation of the HIF-1a subunit of hypoxia-inducible factor 1 (HIF-1) [39]. Tumor suppressor p53 is a regulator of NF-kB repression by the glucocorticoid receptor [40]. However, both control many physiological processes, including cell cycle arrest, DNA repair, death, etc. Some authors have constructed a crosstalk model of the p53- NF-kB network in order to demonstrate that NF-kB upregulates the transcription of p53, whereas p53 attenuates NF-kB transcription [41]. In sea urchins exposure to ultraviolet radiation provokes the down-regulation of p53, leading to apoptosis, as shown by the significant increase in DNA strand breaks in the nuclei of developing embryos [42]. Protein δ-2-catenin is normally expressed in the brain where it is important for normal cognitive development [43]. The catenin-presenilin interaction has implications for cadherin function and regulation of cell-to-cell adhesion [44]. Moreover, *δ-2-catenin* has been implicated as a regulator of the NF-κB transcription factor [45]. *HIF1A* is a heterodimeric transcription factor that regulates cellular energy metabolism and angiogenesis in response to oxygen deprivation. In fact, it functions as a master regulator of cellular and systemic homeostatic response to hypoxia by activating transcription of many genes, including those involved in energy metabolism, angiogenesis, apoptosis, and other genes whose protein products increase oxygen delivery or facilitate metabolic adaptation to hypoxia. This gene thus plays an essential role in embryonic vascularization, tumour angiogenesis and pathophysiology of ischemic disease. HIF1A abundance (and its subsequent activity) is regulated transcriptionally in an NF-κB-dependent manner [46]. Moreover, Ben-Tabou de-Leon et al. [47] demonstrated that the initial activation of aboral genes in sea urchin depends directly on the redox sensitive transcription factor *HIF1A*. Our results show very clearly that all four HUB genes are molecular targets of PUAs, which are able to induce significant variations in the expression of these genes with respect to the controls in a dose-dependent manner (see Fig 7 and S1 Fig). Fig 6 shows that the four HUB genes are interconnected and modulated each other. According this interactomic analysis, we hypothesize that HUB genes in turn could be able to modulate the expression levels of thirteen *P*. *lividus* genes reported in the network (see also Table 2): six stress genes (*hsp70*, *hsp60*, *hsp56*, *MTase*, *cytb*, *p38MAPK*), four genes implicated in development and differentiation (*sox9*, *blimp*, *Alix*, *Wnt6*), and two genes implicated in detoxification processes (*MDR1* and *CAT*).

**Fig 8:**
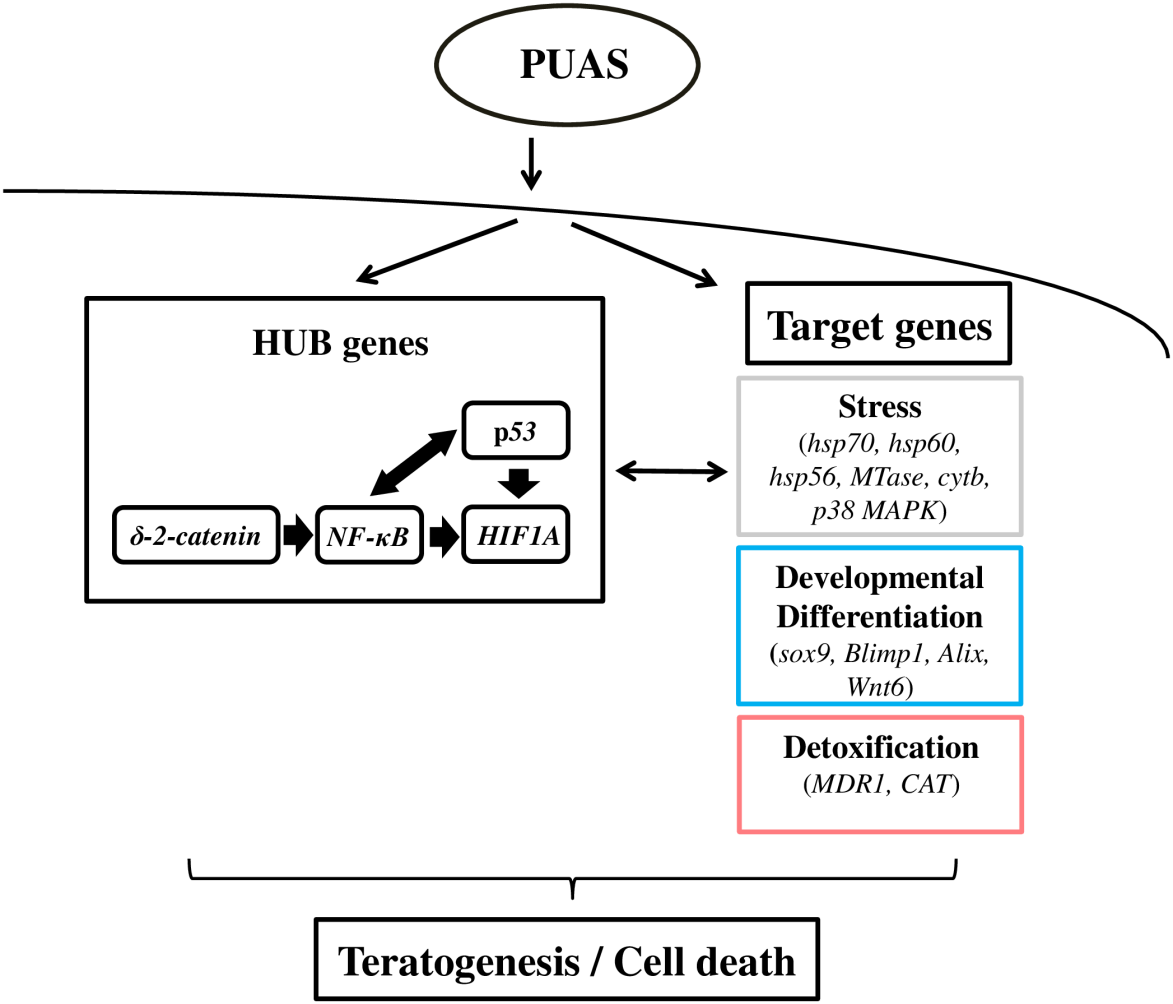
Working model of hypothetical pathways potentially involved in the toxic PUAs stress response. PUAs may be able to activate four HUB genes (δ-2-catenin regulates *NF-κB*, which regulates *HIF1A*, which in its turn is regulated by *p53*; a crosstalk between *p53* and *NF-κB* has been reported in ref. 41) or several genes (belonging to different functional responses). All these genes may induce teratogenesis and/or to apoptosis in the sea urchin embryo.

Fig 8 proposes a working model describing the hypothetical pathways potentially involved in the diatom-derived PUAs stress response in sea urchins.

PUAs initially down-regulated (probably through transmembrane receptors) the four nuclear HUB genes: they initially down-regulated δ-2-catenin, which regulates *NF-κB*, and then *HIF1A*, which in turn is regulated by *p53*; *p53* and *NF-κB* regulate each other. On the other hand, PUAs may also affect several genes, having a key role in different functional responses, such as stress, development, differentiation and detoxification. A crosstalk is possible between HUB genes and target genes. All these genes may drive sea urchin embryos towards teratogenesis and/or apoptosis (or cell death), depending on the PUAs exposure dose, as also shown in Varrella et al. [15].

Summarising, the present work represents the first molecular report of the effects of diatom-derived PUAs on gene networks, using the sea urchin as a bioindicator. We demonstrate how changes in gene expression levels may be used as an early indicator of stressful conditions in the marine environment. In fact, as observed in most adaptive responses, control of gene expression is tightly regulated and has fast response kinetics, which enables the cell to change its transcriptional capacity within minutes in the presence of stress and to return to its basal state after the stress is removed [48]. According to our data it is possible to hypothesize that the three PUAs are not only capable of switching on their target genes (including HUB genes) at certain concentrations, but its mechanism of action could be highly sophisticated. More in details, we probably detected different effects at different PUA concentrations, because the three PUAs could not directly act on their target genes (including HUB genes), but their actions could be mediated through other genes. Hence, future efforts will regard the evaluation of the entire transcriptome and/or proteome to understand what factors, such as mRNAs and/or proteins, can be modulated by the three PUAs at the different concentrations.

Our study shows how marine organisms may attempt to defend themselves from environmental toxicants, benefitting from the protection provided by an integrated network of genes, the defensome [14,49]. Further investigations are needed to better clarify the negative effects at the molecular level of these molecules on benthic organisms, causing deleterious effects during diatom blooms at sea [50].

## Supporting Information

S1 Fig Dose-dependent variation of gene expression levels for the HUB gens. Histograms show A) decadienal, B) heptadienal and C) octadienal dose-dependent variations in expression levels of the four HUB genes. Samples incubated with increasing decadienal (1.0, 1.3, 1.6, 2.0, 2.3 μM), heptadienal (2.0, 2.5, 3.0, 5.5, 6.0 μM) and octadienal (2.5, 4.0, 4.5, 5.0, 7.0, 8.0 μM) concentrations were collected at different stages of development: early blastula (5hp), swimming blastula (9hpf), prism (24hpf) and pluteus (48 hpf) Data are reported as a fold difference (mean ± SD), compared to the control embryos in sea water without aldehydes. Fold differences greater than ±2 (see the dotted horizontal guide lines at the values of +2 and −2) were considered significant.
(PPT)

S1 Table Function for the four new genes analyzed in the present study. (DOC)

S2 Table Data of expression level were reported as a fold difference from control at 5, 9, 24 48 hpf at different aldehyde concentrations: decadienal 1.0, 1.3, 1.6, 2.0, 2.3 μM; heptadienal 2.0, 2.5, 3.0, 5.5, 6.0 μM; octadienal 2.5, 4.0, 4.5, 7.0, 8.0 μM. Fold differences greater than ± 2 were considered significant.
(XLSX)
