# Supplementary material for: Toxic Diatom Aldehydes Affect Defence Gene Networks in Sea Urchins
Source: PLoS One. 2016 Feb 25;11(2):e0149734. doi: 10.1371/journal.pone.0149734 (PMC4767821; doi:10.1371/journal.pone.0149734)
Supplement: S1 Fig — Histograms show A) decadienal, B) heptadienal and C) octadienal dose-dependent variations in expression levels of the four HUB genes. Samples incubated with increasing decadienal (1.0, 1.3, 1.6, 2.0, 2.3 μM), heptadienal (2.0, 2.5, 3.0, 5.5, 6.0 μM) and octadienal (2.5, 4.0, 4.5, 5.0, 7.0, 8.0 μM) concentrations were collected at different stages of development: early blastula (5hp), swimming blastula (9hpf), prism (24hpf) and pluteus (48 hpf) Data are reported as a fold difference (mean ± SD), compared to the control embryos in sea water without aldehydes. Fold differences greater than ±2 (see the dotted horizontal guide lines at the values of +2 and −2) were considered significant. (PPT) [file pone.0149734.s001.ppt]

## Slide 1
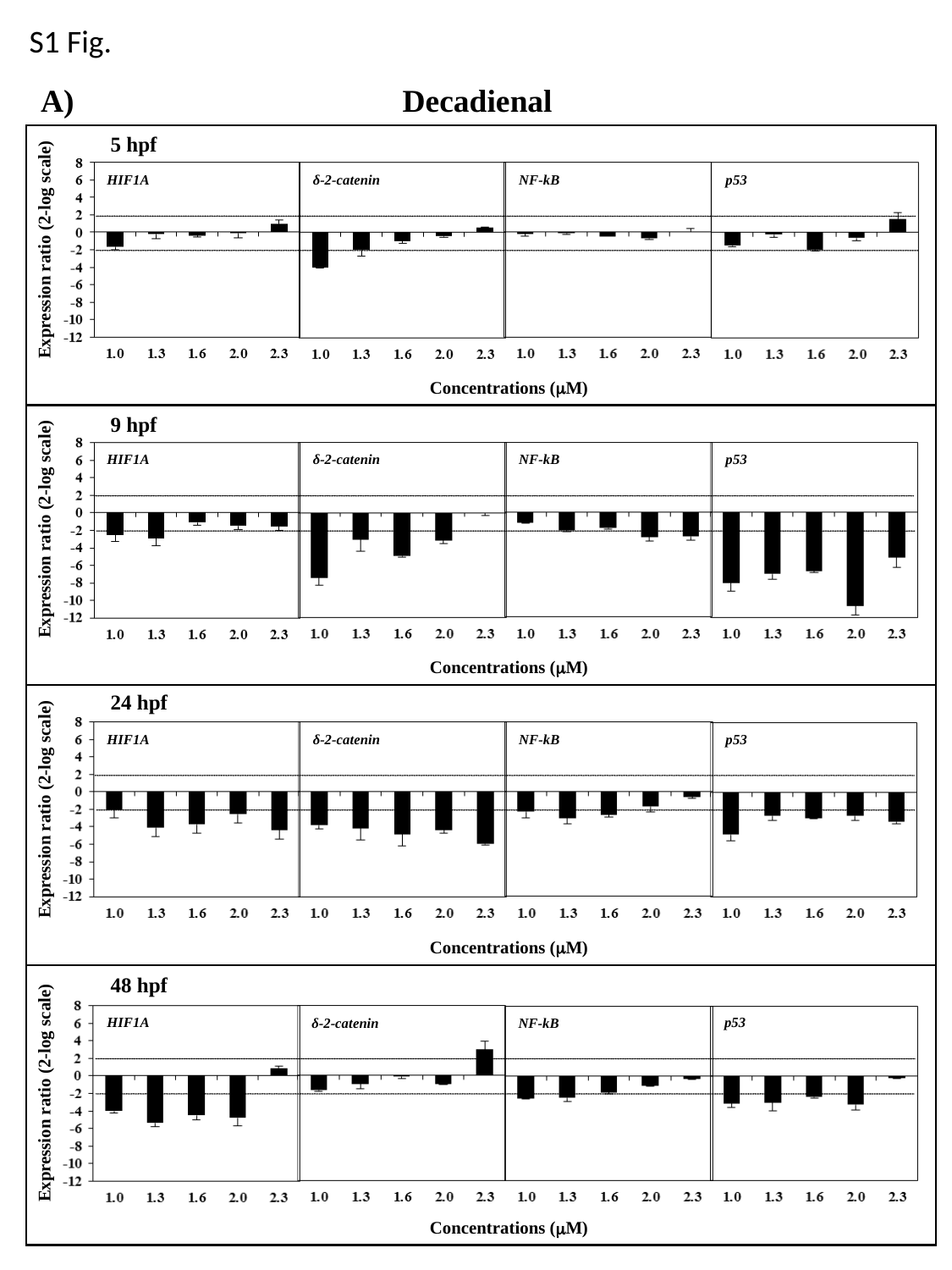

S1 Fig.
A)
Decadienal
5 hpf
δ-2-catenin
NF-kB
p53
HIF1A
Expression ratio (2-log scale)
Concentrations (μM)
9 hpf
δ-2-catenin
NF-kB
p53
HIF1A
Expression ratio (2-log scale)
Concentrations (μM)
24 hpf
δ-2-catenin
NF-kB
p53
HIF1A
Expression ratio (2-log scale)
Concentrations (μM)
48 hpf
HIF1A
p53
NF-kB
δ-2-catenin
Expression ratio (2-log scale)
Concentrations (μM)

## Slide 2
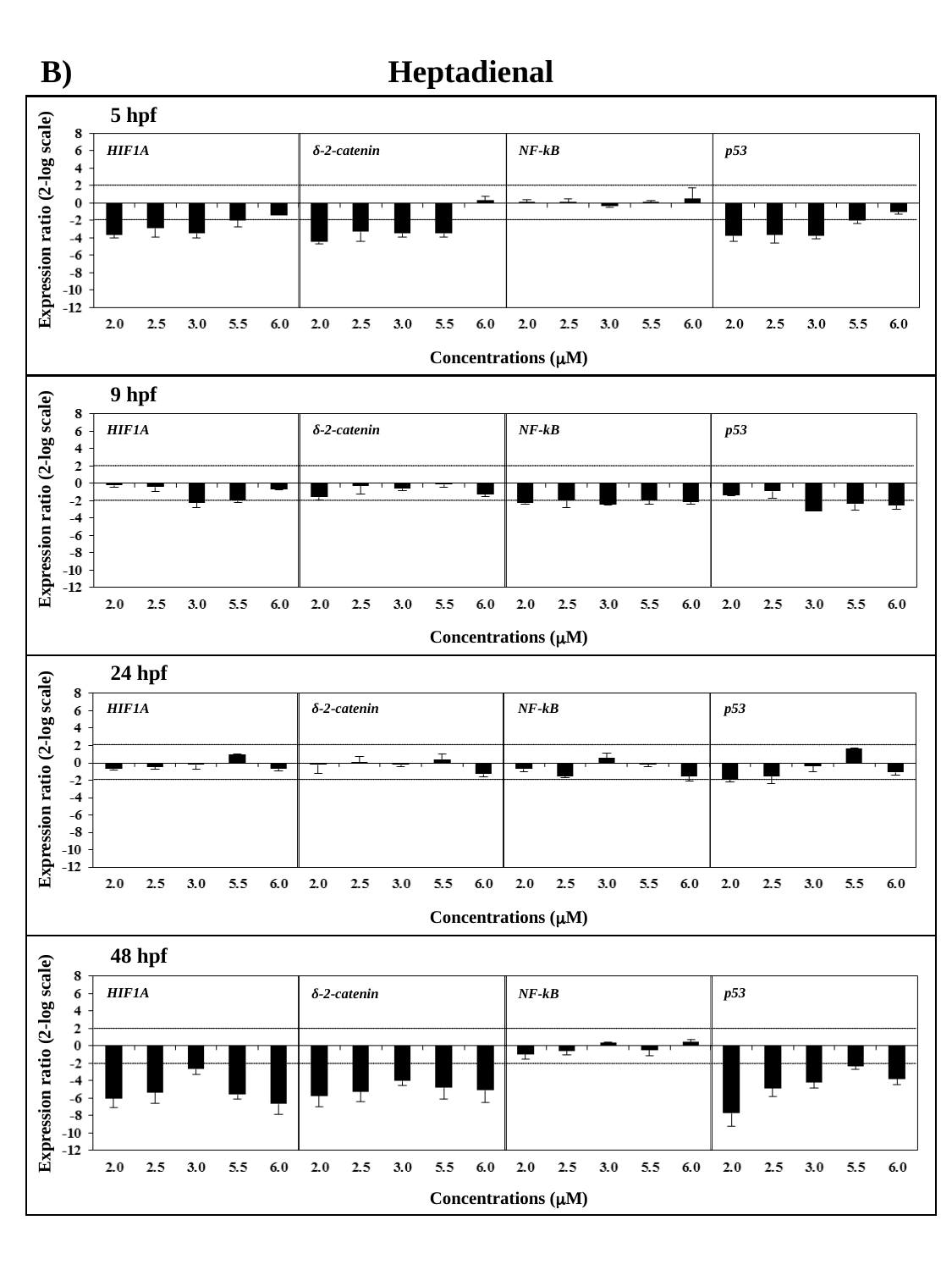

Heptadienal
B)
5 hpf
δ-2-catenin
NF-kB
p53
HIF1A
Expression ratio (2-log scale)
Concentrations (μM)
9 hpf
δ-2-catenin
NF-kB
p53
HIF1A
Expression ratio (2-log scale)
Concentrations (μM)
24 hpf
δ-2-catenin
NF-kB
p53
HIF1A
Expression ratio (2-log scale)
Concentrations (μM)
48 hpf
HIF1A
p53
δ-2-catenin
NF-kB
Expression ratio (2-log scale)
Concentrations (μM)

## Slide 3
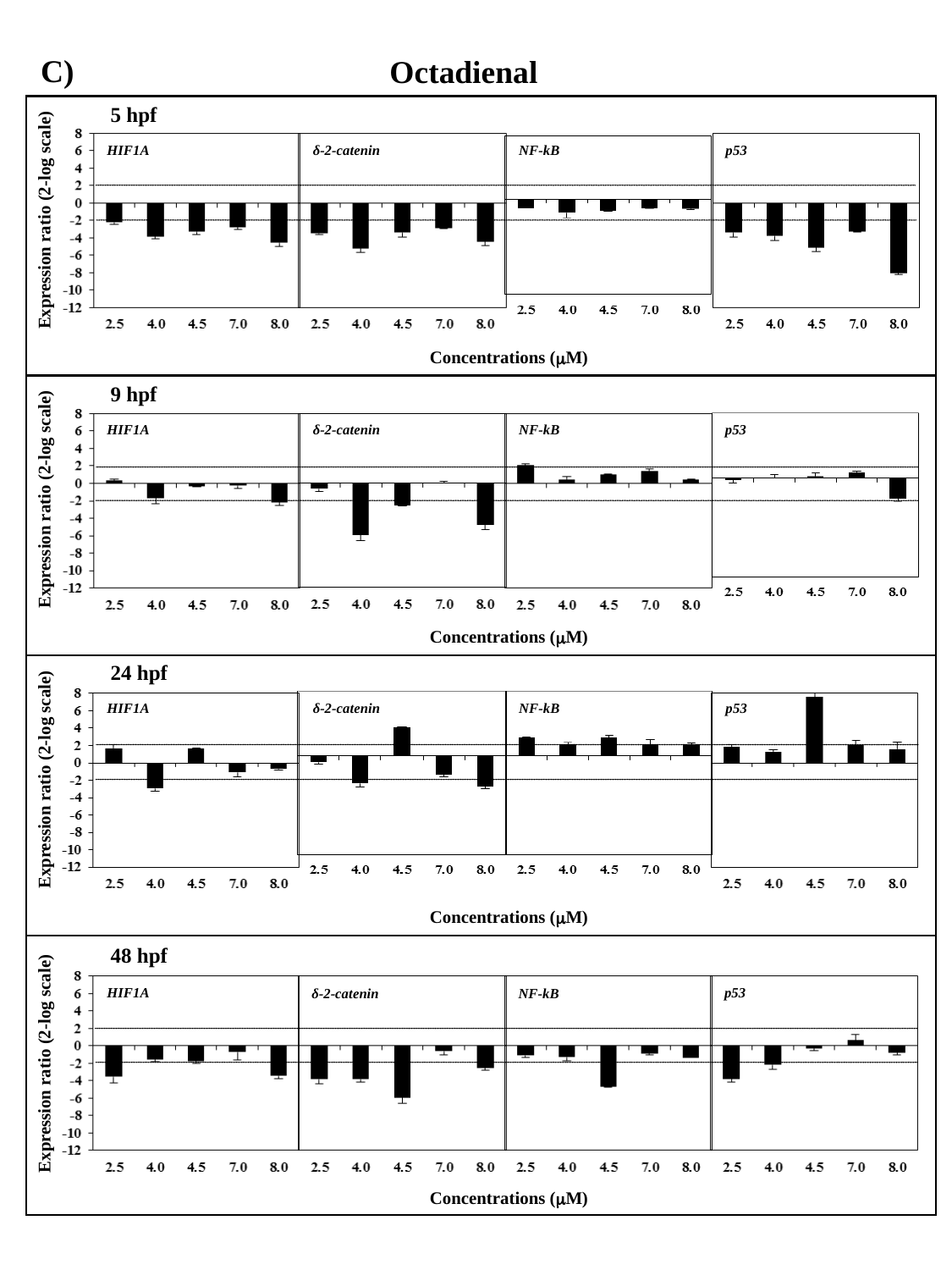

C)
Octadienal
5 hpf
δ-2-catenin
NF-kB
p53
HIF1A
Expression ratio (2-log scale)
Concentrations (μM)
9 hpf
δ-2-catenin
NF-kB
p53
HIF1A
Expression ratio (2-log scale)
Concentrations (μM)
24 hpf
δ-2-catenin
NF-kB
p53
HIF1A
Expression ratio (2-log scale)
Concentrations (μM)
48 hpf
HIF1A
p53
δ-2-catenin
NF-kB
Expression ratio (2-log scale)
Concentrations (μM)
