# Supplementary material for: Toxic Diatom Aldehydes Affect Defence Gene Networks in Sea Urchins
Source: PLoS One. 2016 Feb 25;11(2):e0149734. doi: 10.1371/journal.pone.0149734 (PMC4767821; doi:10.1371/journal.pone.0149734)
Supplement: S1 Table — (DOC) [file pone.0149734.s002.doc]

**S1 Table.** Function for the four new genes analyzed in the present study.

| **Gene** | **Acronym** | **Function** |
| --- | --- | --- |
|  |  |  |
| ***Nuclear factor kappa-light-*** | *NF-κB* | a protein complex that controls transcription of DNA, |
| ***chain-enhancer of activated B cells*** |  | involved in cellular responses to stimuli such as |
|  |  | stress, cytokines, free radicals, ultraviolet irradiation, |
|  |  | and bacterial or viral antigens |
|  |  |  |
| ***Tumor protein p53*** | *p53* | protein that regulates the cell cycle, functioning as a |
|  |  | tumor suppressor, preventing cancer; it has been |
|  |  | described as "the guardian of the genome" because of |
|  |  | its role in conserving stability by preventing genome |
|  |  | mutations |
|  |  |  |
| ***Cadherin-associated protein*** | *Ctnnd2* | protein involved in the coordination of cell-cell |
| ***(catenin) delta 2*** |  | adhesion and gene transcription; this protein is |
|  |  | cells stick together and plays a role in cell movement |
|  |  |  |
| ***Hypoxia inducible factor*** | *HIF1A* | regulator of cellular and systemic homeostatic |
| ***1-alpha*** |  | response to hypoxia by activating transcription of |
|  |  | many genes including those involved in energy |
|  |  | metabolism, angiogenesis, apoptosis, and other genes whose protein products increase oxygen delivery or facilitate metabolic adaptation to hypoxia |
|  |  |  |
